# The Impact of Maternal BMI on the Efficacy and Safety of Oral Misoprostol for Labor Induction

**DOI:** 10.3390/ph18121888

**Published:** 2025-12-14

**Authors:** Maciej W. Socha, Wojciech Flis, Julia Sowińska, Martyna Stankiewicz, Anita Kazdepka-Ziemińska

**Affiliations:** 1 Department of Obstetrics and Gynecology, St. Adalbert’s Hospital, Gdańsk Copernicus Healthcare Entity, Jana Pawła II 50, 80-462 Gdańsk, Poland; 2 Department of Perinatology, Gynecology and Gynecologic Oncology, Collegium Medicum in Bydgoszcz, Nicolaus Copernicus University, Łukasiewicza 1, 85-821 Bydgoszcz, Poland

**Keywords:** misoprostol, induction of labor, pharmacokinetics, obesity, cervical ripening

## Abstract

**Background**: Maternal obesity may influence the efficacy and course of induction of labor (IoL). Misoprostol, a prostaglandin E1 analogue, is widely used for cervical ripening, but evidence regarding its effectiveness in obese women remains limited. This study aimed to evaluate the efficacy and safety of oral misoprostol for IoL across different body mass index (BMI) categories. **Methods**: This prospective study was conducted at a tertiary center. Term singleton pregnancies with medical indications for IoL and an unfavorable cervix (Bishop score < 6) received oral misoprostol 50 μg every 4 h to a maximum of 200 μg. Primary outcomes were vaginal delivery (VD) rates. Secondary outcomes included cesarean section (CS) rate, oxytocin use, labor duration, analgesia, adverse events, and neonatal outcomes. **Results**: Among 291 participants (43.0% overweight; 40.2% obese), the Bishop score increased from 2.3 to 6.2 (*p* < 0.0001). VD occurred in 77.3%, and CS in 22.7%. Most women delivered within 48 h (96.6%). Higher BMI correlated with longer time to contractions, pain onset, and delivery, as well as with more misoprostol doses. Neonatal outcomes were uniformly favorable, with median Apgar scores of 10 at 1, 5, and 10 min, and mean umbilical pH values ranging from 7.2 to 7.3. Adverse events were infrequent, with tachysystole observed in 1.7% of cases. **Conclusions**: Oral misoprostol is an effective and safe IoL method across BMI categories, achieving high vaginal delivery rates and favorable neonatal outcomes. Obesity modestly prolongs induction and increases dose requirements, supporting individualized dosing and close monitoring.

## 1. Introduction

Recent decades have witnessed an epidemic of obesity, which has become a major global health concern. In recent years, the prevalence of overweight and obesity (defined as BMI 25–30 kg/m^2^ and BMI ≥ 30 kg/m^2^, respectively) has risen to alarming rates [1,2]. In Central Europe, estimations of the prevalence of overweight and obesity are particularly concerning, with most countries reporting figures approaching 20% for obesity alone in the general population. Among pregnant women, the prevalence of obesity reaches up to 20% in the European population, and some studies even suggest that by 2025, this percentage will rise to approximately 25% [1,2,3].

Obesity during pregnancy, apart from its negative impact on quality of life, is strongly associated with an increased number of perinatal, obstetric, and neonatal complications such as gestational hypertension, gestational diabetes, and abnormal fetal growth. Moreover, obese pregnant women who have pre-existing obesity-related diseases (e.g., chronic hypertension or diabetes) are at an even higher risk of complications during pregnancy and delivery due to these underlying conditions [1,3,4,5].

Induction of labor (IoL) is one of the most frequently performed procedures in modern obstetrics—approximately 20% of pregnant women undergo IoL, with increasing rates worldwide [6]. It is generally defined as the iatrogenic initiation of uterine contractions leading to a successful vaginal delivery, typically within 24–48 h [7]. In contemporary obstetric practice, the standard protocol for induction of labor includes intravenous oxytocin administration. However, the majority of pregnant women require prior cervical ripening due to an unfavorable cervix. The most common induction protocol therefore consists of a cervical ripening agent followed by intravenous oxytocin infusion. Prostaglandin analogues (such as Misoprostol or Dinoprostone) or mechanical devices (e.g., a Foley catheter) are widely used to prepare the cervix, with varying efficacy [7,8,9,10].

Misoprostol, a prostaglandin E_1_ analogue, is one of the most commonly used pharmacological agents for cervical ripening. Its efficacy and clinical safety have been confirmed in numerous studies [10,11,12]. This drug is highly valued for its versatility, as it can be administered via several routes (vaginal, oral, or buccal) and has demonstrated high effectiveness. Oral Misoprostol is particularly well accepted by patients due to its minimally invasive nature. The pharmacological effect of Misoprostol is primarily dose-dependent [13,14,15].

Considering that obesity significantly influences many aspects of pregnancy and childbirth, it seems reasonable to assume that it may also affect the course and outcomes of labor induction. Indeed, several studies have indicated that IoL in obese women is associated with an increased risk of induction failure [16,17,18]. However, there is currently no consensus on which induction method is most effective and clinically safe for obese pregnant patients [2,18].

Therefore, the primary objective of this study was to evaluate the clinical efficacy of oral misoprostol for the induction of labor in women with term pregnancies, with particular emphasis on its effectiveness in patients with elevated body mass index (BMI). The secondary objective was to assess the safety profile of the intervention by analyzing maternal and neonatal outcomes, including blood loss, neonatal condition and possible adverse effects. The findings may contribute to optimizing induction protocols and improving perinatal outcomes in this growing population of patients.

## 2. Results

The tables below present the general obstetric outcomes following oral Misoprostol pre-induction. In the subsequent sections, more detailed results are presented in a correlational analysis according to the patients’ BMI categories within the study group.

The mean age of participants was 30.4 years (range: 18–46), and the mean BMI was 29.3 kg/m^2^ (range: 20.5–51.4). The average body weight was 81.2 kg (range: 59.4–140.0). Most labor inductions (76%) occurred between 39 and 41 weeks of gestation. Inductions at 37, 38, and 42 weeks accounted for 3.8%, 11.0%, and 9.3% of cases, respectively (Table 1).

Regarding parity, 71.8% of women were nulliparous and 28.2% were multiparous. In terms of BMI categories, 16.8% had a normal BMI (18.5–24.9), 43.0% were overweight (25.0–29.9), and 40.2% were obese (BMI ≥ 30). Within the obese group, 33.0% had class I obesity, 5.5% class II, and 1.7% class III (morbid obesity).

The leading reasons for IoL were post-term pregnancy (35.1%), large for gestational age (21.3%) and pregnancy-induced hypertension (12.2%). Other, less common included fetal growth restriction (8.2%), oligohydramnios (7.7%) and gestational diabetes-type 2 (6.3%). Rare causes such as intrahepatic cholestasis, polyhydramnion (both 1.4%), maternal age over 40 (0.7%) and small for gestational age (0.3%) were also noted.

The mean initial Bishop score (Table 2) prior to Misoprostol administration was 2.3 ± 1.1 (range: 0–5; median: 2.0; 95% CI: 2.2–2.5). Following oral Misoprostol induction, the mean Bishop score increased to 6.2 ± 2.3 (range: 1–12; median: 6.0; 95% CI: 6.0–6.5). Surprisingly, the Bishop score showed a marked and statistically significant rise after Misoprostol was administered (*p* < 0.0001).

On average, patients received 3.1 ± 1.1 doses of oral Misoprostol (median: 3.0; range: 1–4; 95% CI: 2.9–3.2). Over half of the participants (52.6%) reached the full four-dose protocol, while 47.4% responded to fewer doses: 39.8% needed only three doses, 4.5% responded after two, and 3.1% after just one (Table 3). The most common reason for discontinuing Misoprostol was the onset of regular uterine contractions (43.4%). In fewer cases (4.1%), the protocol was halted due to pre-labor rupture of membranes (PROM).

The majority of patients delivered vaginally (*n* = 225, 77.3%), while 66 patients (22.7%) required a cesarean section (CS) (Table 4).

The majority of deliveries occurred within 24 h (*n* = 181, 62.2%). A further 100 deliveries (34.4%) took place more than 24 h but less than 48 h after IoL initiation, while only 10 cases (3.4%) required more than 48 h to complete the delivery process (Table 5).

Among the 181 deliveries that occurred within the first 24 h, 145 (64.4%) were vaginal, while 36 (54.5%) were cesarean sections. In the 24–48 h time frame, 74 vaginal deliveries (32.9%) and 26 cesarean sections (39.4%) were recorded (Table 6). Deliveries occurring beyond 48 h were rare—6 vaginal (2.7%) and 4 cesarean (6.1%), accounting for a total of 10 cases (3.4% of all deliveries).

The vast majority of operative deliveries occurred within 24 h (*n* = 11, 91.6%), while only one case (8.4%) occurred within 24–48 h. No operative deliveries were recorded beyond 48 h (Table 7).

Within the first 24 h, 36 cesarean sections were performed. The dominant indication was impending fetal asphyxia (*n* = 35, 97.2%), while one case (2.8%) was due to lack of labor progression (Table 8). Within 24–48 h, 26 cesarean sections were performed—again, most commonly due to impending fetal asphyxia (*n* = 22, 84.6%), followed by lack of labor progression (*n* = 4, 15.4%). Beyond 48 h, four cesarean sections were recorded. In this subgroup, the majority (*n* = 3, 75%) were performed due to lack of labor progression, while one case (25%) was associated with impending fetal asphyxia.

Most deliveries across all BMI categories occurred within 24 or 48 h. In normal-weight patients, 71.4% delivered within 24 h. Among overweight and class I obese women, 63.2% and 60.4% delivered within 24 h, while class II and III obese patients more often delivered within 24–48 h. There was no significant association between BMI and delivery timing (*p* > 0.05).

A significant association was found between BMI and the proportion of vaginal deliveries within 24 h (*p* = 0.032). In lower BMI categories (normal, overweight, class I), most vaginal deliveries occurred within 24 h. In contrast, among class II–III obese patients, the majority occurred within 24–48 h (Table 9).

Cesarean deliveries mostly occurred within 24 or 24–48 h across all groups, with no statistically significant difference between BMI categories (*p >* 0.05).

Oxytocin administration increased with BMI: from 28.6% in normal-weight women to 60% in class III obesity. However, the trend was not statistically significant (*p* > 0.05).

The median duration of the first stage of labor ranged from 3.0 to 6.5 h, slightly longer in class III obesity. The second stage was comparable across all BMI groups (0.8–1 h). No significant differences were observed in labor duration (*p* = 0.1892 and *p* = 0.4269, respectively).

The overall use of intrapartum analgesia remained consistent across all BMI categories. Among women with normal BMI (18.5–24.9), epidural anesthesia was most frequently chosen (36.7%), followed by intravenous remifentanil (24.5%). In overweight patients (BMI 25–29.9), remifentanil slightly outpaced epidural use (30.4% vs. 24.8%). Class I obesity (BMI 30–34.9) showed a preference shift back toward epidural analgesia (29.2%), with remifentanil used by 25.0% of patients. In contrast, remifentanil dominated in class II obesity (BMI 35–39.9), used by 43.8% compared to 18.8% who received an epidural. Interestingly, patients with class III obesity (BMI ≥ 40) favored epidural analgesia again (60.0%), with only 20.0% choosing remifentanil. No statistically significant association was found between BMI and the type of analgesia administered (*p* = 0.525 and *p* = 0.225).

As for uterine hyperstimulation (tachysystole), no cases were reported in normal-weight or class III obese patients. Isolated cases appeared among overweight (1.6%) and class I obese (1.0%) women, but the highest incidence was observed in the class II obesity group (12.5%). This association was statistically significant (*p* = 0.015, Chi-square test). Importantly, no other adverse effects related to Misoprostol were identified across the study population.

The time to onset of regular contractions was comparable among normal-weight, overweight, and class I obese women—averaging 15.0, 16.8, and 16.3 h, respectively. However, for women with class II and III obesity, the time increased noticeably to 21.5 and 25.1 h (Table 10).

A similar pattern was seen for pain onset. In lower BMI groups, pain began around 15–17 h, while in higher obesity classes, it was delayed to 21.8 and 25.1 h, mirroring the timing of contractions.

The mean time to delivery also increased slightly with BMI. In the normal-weight, overweight, and class I obese groups, it was 19.3, 21.9, and 21.5 h, respectively, while in class II and III obesity groups, it was 26.4 and 31.5 h.

Interestingly, intrapartum blood loss remained relatively consistent across groups, ranging from ~290 mL to ~340 mL, with no meaningful differences by BMI.

Regarding perineal injuries, 67.4% of normal-weight women experienced some form of perineal trauma, most commonly first- (51.5%) and second-degree (45.5%) tears. In overweight patients, 62.4% sustained perineal injuries, predominantly of the second degree (55.1%). In class I obesity, perineal injuries were noted in 57.3% of cases, with second-degree tears being most common (65.5%). In class II obesity, perineal injuries were observed in 56.2% of patients, and in class III obesity, only 40% of patients experienced any perineal trauma, limited to second-degree tears.

No statistically significant differences were found between BMI groups regarding time to regular contractions, time to pain onset, time to delivery, total blood loss, or the occurrence and degree of perineal injuries (*p* = 0.087, *p* = 0.077, *p* = 0.058, *p* = 0.708, and *p* = 0.670, respectively).

Across all BMI categories, newborns scored a median of 10 points on the Apgar scale at 1, 5, and 10 min of life—indicating excellent neonatal condition regardless of maternal weight. No statistically significant differences were observed (*p* = 0.088, 0.715, and 0.623, respectively).

Blood gas analysis showed similar trends. The mean umbilical artery pH was 7.2 in normal-weight and overweight women, and 7.3 in all obesity subgroups. The umbilical vein pH remained stable at 7.3 across all groups. Again, no statistically significant associations were found between BMI and neonatal blood pH (*p* = 0.567 for artery and *p* = *0.5442* for vein), confirming consistent acid–base balance across the board (Table 11).

Higher BMI was significantly associated with longer labor dynamics and increased medication needs. Specifically, increasing BMI positively correlated with: time to onset of uterine contractions (*R* = 0.13, *p* = 0.025), time to pain onset (*R* = 0.14, *p* = 0.019), total time to delivery (*R* = 0.15, *p* = 0.013), number of Misoprostol doses needed for cervical ripening (*R* = 0.15, *p* = 0.013), and—unexpectedly—slightly higher umbilical vein pH values (*R* = 0.20, *p* = 0.0005).

In contrast, a higher BMI was linked to a lower Bishop score after Misoprostol administration (*R* = –0.17, *p* = 0.005), suggesting a less favorable cervical response. However, no significant correlations were found between BMI and: duration of the first or second stage of labor, perinatal blood loss, Apgar scores at 1, 5, or 10 min, umbilical artery pH and initial Bishop score before induction (Table 12).

## 3. Discussion

As mentioned earlier, approximately one in five women undergoes IoL for fetal or maternal indications. Although intravenous oxytocin infusion remains the gold standard for IoL due to its high efficacy in initiating uterine contractions, it has a limited effect on cervical ripening. Therefore, effective and safe labor induction requires adequate pre-induction cervical preparation. The likelihood of successful vaginal delivery increases when the Bishop score exceeds 6 points [19,20]. Consequently, in patients with medical indications for IoL and an unfavorable cervix (Bishop score < 6), pre-induction of labor is strongly recommended [18,21,22,23].

In modern obstetrics, several pre-induction methods are available, differing in safety and efficacy profiles. Misoprostol, a prostaglandin E1 analogue, can be administered orally or vaginally. The oral route is generally preferred due to its non-invasive nature and high patient acceptability, with comparable efficacy to mechanical methods such as the Foley catheter [24,25]. The primary objective of the present study was to evaluate the clinical efficacy and safety of oral Misoprostol for labor induction in term pregnancies, with a particular focus on patients with varying degrees of obesity. While previous studies have explored the use of misoprostol in obese pregnant women, the current study adds several important dimensions to the existing literature. First, we provide a detailed time-based stratification of delivery outcomes (within 24 h, 24–48 h, and >48 h), allowing for a more granular analysis of induction efficacy in relation to maternal BMI. Second, we assess not only the overall success of labor induction, but also the specific modes of delivery—including cesarean section and instrumental vaginal birth—across BMI subgroups. Third, our data are derived from a real-world clinical population, which strengthens the generalizability of the findings and offers practical insight for obstetric management in diverse settings. Taken together, these contributions may help to fill existing gaps in knowledge regarding the interaction between maternal obesity and the clinical effectiveness and safety profile of oral Misoprostol.

The results clearly demonstrated the effectiveness of the adopted regimen. The vast majority of patients (77.3%) achieved vaginal delivery after Misoprostol pre-induction, while only 22.7% required cesarean section. These outcomes meet the established definition of successful induction of labor and align with previously published findings [26,27]. Furthermore, the observed cesarean section rate remained consistent with international recommendations suggesting an acceptable operative delivery rate of around 20% [28]. These results demonstrate that oral Misoprostol is effective in promoting vaginal delivery without increasing the overall cesarean section rate.

Cervical ripening following Misoprostol administration was satisfactory, with a mean Bishop score of 6.2 points. This finding confirms the ability of Misoprostol to effectively stimulate cervical remodeling, in agreement with previous reports [29,30]. However, a negative correlation between BMI and post-misoprostol Bishop score (R = –0.17, *p* = 0.005) suggests that increased BMI may reduce the cervical response to prostaglandin stimulation. This observation may indicate that adiposity-related hormonal, inflammatory, or receptor-level changes could influence cervical tissue responsiveness.

In nearly half of the patients (47.4%), the full four-dose Misoprostol regimen was not required, as regular uterine contractions began before the final dose. The primary reason for discontinuing the drug was the development of regular uterine activity, confirming the effective uterotonic properties of Misoprostol. This finding reflects the pharmacodynamic mechanism of the drug, which stimulates E-type prostanoid receptor 1 and 2 (EP1 and EP2)—responsible for smooth muscle contraction and cervical softening [31]. Additionally, Misoprostol enhances endogenous prostaglandin E2 release, further facilitating labor onset [31]. Despite the overall high efficacy, a weak but statistically significant positive correlation was found between BMI and the total number of Misoprostol doses administered (R = 0.15, *p =* 0.0134). This suggests a potential trend indicating that women with higher BMI may require slightly higher cumulative doses to achieve a comparable cervical response.

When considering the overall time to delivery, most patients (96.5%) delivered within 48 h, and 62.2% delivered within the first 24 h after initiation of pre-induction. Only 3.4% of patients delivered after more than 48 h, which further confirms the effectiveness of the protocol used. Most cesarean sections performed within 24 or 24–48 h were indicated for impending fetal asphyxia (97.2% and 84.6%, respectively), while those beyond 48 h were mainly due to lack of labor progression (75%). These results are consistent with other studies demonstrating that Misoprostol shortens the time to delivery and achieves high success rates within 24–48 h [13,32,33].

Interestingly, although the observed correlations were weak in magnitude, statistically significant positive relationships were found between BMI and the time to onset of regular uterine contractions (R = 0.13, *p* = 0.025), time to pain onset (R = 0.14, *p* = 0.019), and overall time to delivery (R = 0.15, *p* = 0.013). These low correlation coefficients indicate only a modest linear association. However, the consistency of the trend across multiple labor-related timepoints may suggest that higher BMI is associated with a slightly prolonged course of induction. While these findings do not imply a strong predictive value, they may reflect subtle physiological or pharmacokinetic differences in the response to induction of labor among women with higher body mass.

One possible explanation for this observation lies in the underlying pharmacological and physiological mechanisms associated with obesity. In obese patients, oral Misoprostol may have a larger volume of distribution and slower absorption due to altered gastrointestinal physiology. Moreover, obesity is associated with chronic low-grade inflammation and dysregulation of drug transporters, which may reduce drug bioavailability [34,35]. Given these pharmacological considerations, future studies should explore pharmacokinetic modeling of misoprostol in obese populations. Such modeling may help determine optimal dosing intervals, peak plasma concentrations, and drug half-life across BMI categories [36,37]. A physiologically based pharmacokinetic approach could be particularly valuable in predicting drug absorption and response in women with obesity-related metabolic alterations. Moreover, comparative trials evaluating alternative dosing regimens (e.g., higher initial loading doses or shorter intervals between doses) in obese patients may lead to more tailored and effective induction protocols. These strategies could improve time-to-delivery outcomes without compromising safety.

Despite these temporal differences, the overall efficacy of Misoprostol remained high across all BMI groups. There were no statistically significant differences in total delivery rates (vaginal versus cesarean) between BMI categories, confirming that Misoprostol remains effective even in obese patients. Notably, women with normal or slightly elevated BMI achieved a higher proportion of vaginal deliveries within 24 h, suggesting that this subgroup may experience a faster pharmacologic response. However, patients with obesity ultimately achieved comparable vaginal delivery rates, albeit over a longer induction period.

Oxytocin use for labor augmentation did not differ significantly between BMI groups, although a mild upward trend was observed in women with higher BMI. This observation may reflect slower cervical response to prostaglandins rather than true oxytocin resistance. On the other hand, these differences may also result from significantly different study group sizes, which could have translated into a proportional change in the percentage of oxytocin use.

Similarly, the duration of the first and second stages of labor remained within expected physiological limits, with a non-significant tendency toward a slightly prolonged first stage in women with higher BMI [38,39]. Importantly, despite these minor variations, the induction regimen did not prolong the active phases of labor compared to standard expectations, further confirming the safety and predictability of oral Misoprostol. These data are consistent with previously published studies, and further support the clinical efficacy and predictability of oral Misoprostol across BMI categories [13,32,40].

This study also analyzed the use of intrapartum analgesia. No significant association was found between BMI and the type or frequency of analgesia used. Both remifentanil and epidural anesthesia were applied with comparable frequency across BMI groups. This finding suggests that Misoprostol pre-induction does not increase labor-related pain intensity. Although previous reports have linked prostaglandin-based pre-induction with higher pain scores it should be emphasized that pain is a subjective and multidimensional experience [41,42].

With respect to maternal safety, perineal lacerations were distributed similarly across BMI groups. First- and second-degree tears predominated, consistent with the expected pattern in vaginal deliveries [43,44]. Severe (third-degree) tears were rare and clinically acceptable. Blood loss during delivery was comparable among all BMI categories and did not exceed physiological limits. Misoprostol use was not associated with increased bleeding, which corresponds with the low incidence of perineal trauma. Hyperstimulation (tachysystole) was rare and clinically insignificant overall, though two cases were noted among women with class II obesity (*p* = 0.015). Given the small number of obese patients in this subgroup (*n* = 16), this statistical difference likely reflects sample size imbalance rather than a true clinical effect. These findings contrast with previous studies reporting higher rates of hyperstimulation with Misoprostol use, yet they reinforce the good safety profile of the oral route when properly dosed and monitored [33,45].

Neonatal outcomes further confirmed the safety of the protocol. Apgar scores at 1, 5, and 10 min were uniformly high across all BMI groups, and no statistically significant differences were detected. Similarly, umbilical artery and vein blood gas values remained within normal limits, with mean pH values of 7.2–7.3, regardless of maternal BMI. These data suggest that oral Misoprostol does not adversely affect neonatal condition or acid–base balance at birth. Several limitations of this study should be acknowledged. Firstly, this study was conducted in a single tertiary center, which may limit the generalizability of the findings. Secondly, although the overall sample size (*n* = 291) was sufficient for the main analyses, the subgroups of patients with class II and III obesity were relatively small, which could have affected statistical power for detecting rare complications such as hyperstimulation or cesarean indications. Thirdly, pain intensity and patient satisfaction were not evaluated using standardized tools, which restricts conclusions regarding the subjective experience of labor induction. Additionally, potential confounding factors such as parity, fetal weight, or gestational comorbidities were not analyzed as independent variables. Future multicenter studies with larger sample sizes and standardized outcome measures are warranted to confirm these observations. Despite these limitations, the consistency of the results across parameters supports the robustness of the findings.

## 4. Materials and Methods

### 4.1. Study Design

This was a retrospective clinical control study. The data presented in this paper are part of a larger research project conducted by our team on pre-induction and induction methods of labor. The clinical study was carried out between November 2023 and February 2024 at the Department of Obstetrics and Gynecology, St. Adalbert’s Hospital in Gdańsk, Poland (a tertiary referral center). The study protocol was approved by the Bioethics Committee at the District Medical Chamber in Gdańsk (approval No. KB–46a/23). Written informed consent was obtained from each participant prior to enrollment. All participants were adults (>18 years old).

### 4.2. Study Population

This study included patients with full-term pregnancies (>37 weeks of gestation) who had medical indications for IoL. The indications for IoL were in accordance with the current recommendations of the Polish Society of Gynecologists and Obstetricians [18]. Eligible participants were women qualified for induction of labor due to either maternal or fetal indications and with an unprepared cervix (Bishop score < 6). None of the patients showed signs of labor at the time of induction.

Inclusion criteria were: maternal or fetal indications for IoL, cephalic fetal presentation, singleton live pregnancy, full-term gestation (>37 weeks), and no previous surgical procedures involving the uterine wall.

Exclusion criteria included: premature rupture of membranes (PROM), onset of spontaneous labor, non-cephalic fetal position, and any contraindications to IoL or vaginal delivery according to Polish IoL guidelines [18].

Patients who met the inclusion criteria and did not meet any exclusion criteria were enrolled in this study. During qualification, data were collected on pregnancy progression (confirmed by first-trimester ultrasound), its course, and obstetric history including previous pregnancies and deliveries. Demographic data and relevant medical history were also obtained. During the qualification process for induction of labor, a thorough assessment of maternal and fetal well-being was conducted in every case. Any signs of maternal decompensation or fetal distress were considered absolute contraindications to labor induction. Therefore, patients presenting with compromised maternal or fetal status were not eligible for labor induction and were consequently not included in the study population.

### 4.3. Interventions

Each patient underwent a physical and obstetric examination, which included assessment of cervical status using the Bishop score. This scoring system evaluates five key parameters of the cervix and fetal station: cervical dilation, effacement (length), consistency, position, and the station of the presenting fetal part in relation to the ischial spines. Each parameter is assigned a score from 0 to 2 or 3, and the total score ranges from 0 to 13 [22]. A higher Bishop score indicates a more favorable cervix for induction of labor.

Additionally, and ultrasound scan was performed to assess fetal position and well-being. evaluating fetal position and well-being. Following a positive qualification for both induction of labor and participation in this study, patients were assigned to labor induction using oral prostaglandin E_1_ analogue (Misoprostol).

The pre-induction regimen consisted of oral administration of Misoprostol tablets containing 50 μg of the active substance every 4 h, up to a maximum dose of 200 μg (i.e., no more than four doses) [11]. The regimen was discontinued in the following situations:

- Onset of regular uterine contractions;
- PROM;
- Administration of the maximum total dose (200 μg).

**Figure 1:**
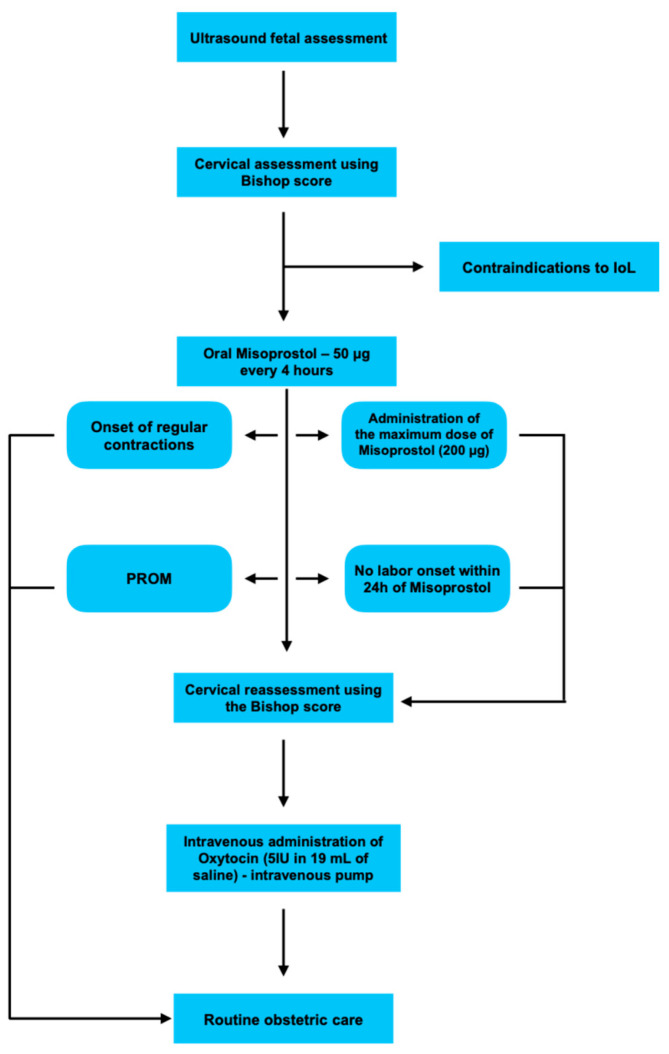
Flowchart illustrating the applied labor induction protocol.

After completion of Misoprostol administration, cervical status was reassessed using the Bishop score. If regular contractions did not occur within 24 h, patients were qualified for induction with intravenous oxytocin infusion. Oxytocin was administered in a solution of 5 IU in 19 mL of saline (0.9% NaCl) via an intravenous infusion pump (Figure 1). Throughout hospitalization, patients were under continuous obstetric supervision.

The following parameters were recorded: labor progression, duration of labor, time from Misoprostol administration to the onset of contractions, perineal injuries, delivery mode (including indications for cesarean section), and any adverse effects of the induction method. Delivery timing and mode of delivery were analyzed based on standardized time intervals following the initial administration of Misoprostol. Vaginal deliveries, cesarean sections, and instrumental vaginal births (performed using obstetrical forceps or vacuum extraction) were categorized based on the duration from Misoprostol administration: within the first 24 h, between 24 and 48 h, and after 48 h. These intervals were consistently applied in both text and tabular data presentation.

Adverse effects included uterine hyperstimulation (tachysystole), nausea, vomiting, and fever. Tachysystole was diagnosed based on the following criteria: uterine contraction lasting > 2 min, absence of relaxation between contractions, or >5 contractions within 10 min. In cases of hyperstimulation, an intravenous infusion of 25 μg of Fenoterol was administered [18].

To assess the degree of obesity, BMI classification was used. Normal weight was defined as a BMI between 18.5 and 24.9 kg/m^2^, overweight as BMI ≥ 25, and obesity as BMI ≥ 30 [2]. Obesity was further subdivided into three classes:

- Class I (BMI 30.0–34.9);
- Class II (BMI 35.0–39.9);
- Class III (morbid obesity, BMI ≥ 40).

During labor, intrapartum analgesia was provided upon request, either as intravenous Remifentanil infusion or epidural anesthesia. Neonatal condition was assessed by umbilical cord blood gas analysis and Apgar score at 1, 3, and 5 min after birth. The Apgar score is a standardized method used to evaluate the physical condition of a newborn immediately after delivery [46]. It includes five criteria: heart rate, respiratory effort, muscle tone, reflex irritability, and skin coloration. Each of these parameters is scored from 0 to 2, resulting in a total score ranging from 0 to 10. Higher scores reflect better neonatal status, with a score of 7–10 generally considered normal, 4–6 moderately abnormal, and 0–3 critically low, requiring immediate resuscitative efforts [46].

### 4.4. Outcomes

The primary outcome was the time from the start of Misoprostol administration to delivery (vaginal or cesarean) and the rate of vaginal delivery in relation to maternal BMI. Secondary outcomes included the proportion of cesarean deliveries, oxytocin use, intrapartum analgesia requirement, and other labor-related parameters.

All collected data were stored electronically using Microsoft Excel and Word software and subsequently subjected to statistical analysis.

### 4.5. Statistical Analysis

All statistical calculations were carried out using the statistical software package StatSoft, Inc., Tulsa, OK, USA (2014). STATISTICA (data analysis software system), version 12.0, www.statsoft.com, and Microsoft Excel spreadsheet.

Quantitative variables were described using the arithmetic mean, standard deviation, median, minimum and maximum values (range), and 95% confidence intervals (95% CI). Qualitative variables were presented as frequencies and percentages.

The Shapiro–Wilk W test was used to assess whether a quantitative variable followed a normal distribution. To test the hypothesis of equal variances, Levene’s test (Brown-Forsythe modification) was applied. The significance of differences between two groups (independent variables model) was assessed using Student’s *t*-test or the Mann–Whitney U test. The Chi-square test of independence was used for qualitative variables. A significance level of *p* = 0.05 was adopted for all statistical calculations.

## 5. Conclusions

In conclusion, oral Misoprostol proved to be an effective and safe method of pre-induction and induction of labor in term pregnancies. Its use was associated with high rates of vaginal delivery, favorable cervical ripening, and excellent maternal and neonatal safety profiles. Although a higher BMI was correlated with a longer induction-to-delivery interval and a greater number of Misoprostol doses, overall success rates and neonatal outcomes remained unaffected. These findings indicate that oral Misoprostol can be effectively used across BMI categories, though individualized dosing and careful monitoring may optimize outcomes, particularly in obese patients.

## Figures and Tables

**Table 1:** Demographic data of the study group.

		Study Group (*n* = 291)
**Age (years)**		
Mean		30.4
Range		18.0–46.0
median (IRQ)		31.0 (6.0)
95%CI		[29.9; 30.9]
**Weight (kg)**		
Mean		81.2
Range		59.4–140.0
median (IRQ)		80.0 (17.0)
95%CI		[79.6; 82.7]
**Age of gestation (weeks)**		
37		11 (3.8%)
38		32 (11.0%)
39		77 (26.5%)
40		63 (21.6%)
41		81 (27.8%)
42		27 (9.3%)
**Parity**		
Nulliparous		209 (71.8%)
Multiparous		82 (28.2%)
**BMI (kg/m^2^)**		
Normal weight	18.5–25	49 (16.8%)
Overweight	25–30	125 (43.0%)
Obesity class I	30–34.9	96 (33.0%)
Obesity class II	35–39.9	16 (5.5%)
Obesity class III	>40	5 (1.7%)
**BMI (kg/m^2^)**		
Mean		29.3
Range		20.5–51.4
median (IRQ)		29.0 (5.8)
95%CI		[28.8; 29.8]
**Indications for IoL**		
Post-term pregnancy		102 (35.1%)
Large for gestational age (LGA)		62 (21.3%)
Pregnancy induced hypertension (PIH)		37 (12.2%)
Fetal growth restriction (FGR)		24 (8.2%)
Oligohydramnios		22 (7.7%)
Gestational diabetes mellitus type 2 (GDM G2)		18 (6.3%)
Gestational diabetes mellitus type 1 (GDM G1)		15 (5.4%)
Intrahepatic cholestasis of pregnancy (ICP)		4 (1.4%)
Polyhydramnios		4 (1.4%)
Maternal Age > 40 Years		2 (0.7%)
Small for gestational age (SGA)		1 (0.3%)

IoL: Induction of labor.

**Table 2:** Characteristics of the study group in terms of the Bishop cervical score (BS).

	Group (*n* = 291)	*p*-Value
**Initial BS**		0.704 ^1^
mean (SD)	2.3 (1.1)	
range	0.0–5.0	
median (IRQ)	2.0 (1.0)	
95%CI	[2.2; 2.5]	
**BS after oral Misoprostol**		<0.0001 ^1^
mean (SD)	6.2 (2.3)	
range	1.0–12.0	
median (IRQ)	6.0 (3.0)	
95%CI	[6.0; 6.5]	

^1^ U Mann–Whitney.

**Table 3:** Characteristics of the group in terms of number of doses and reasons for discontinuation of oral Misoprostol administration.

	Group (*n* = 291)
**Overall**	
mean (SD)	3.1 (1.1)
range	1.0–4.0
median (IRQ)	3.0 (2.0)
95%CI	[2.9; 3.2]
**Misoprostol doses**	
**1**	9 (3.1%)
**2**	13 (4.5%)
**3**	116 (39.8%)
**4**	153 (52.6%)
**Reason for discontinuation**	
Uterine contractions	126 (43.4%)
PROM	12 (4.1%)

PROM (pre-labour rupture of membranes).

**Table 4:** Characteristics of the study group in terms of the percentage of vaginal deliveries and cesarean deliveries.

Type of Delivery	Group (*n* = 291)
**Vaginal delivery (VD)**	225 (77.3%)
**Cesarean section (CS)**	66 (22.7%)

**Table 5:** Characteristics of the study group in terms of deliveries within 24 h, >24–48 h, and over 48 h.

Delivery (Vaginal and Cesarean)	Group (*n* = 291)
**24 h**	
	181 (62.2%)
**>24–48 h**	
	100 (34.4%)
**>48 h**	
	10 (3.4%)

**Table 6:** Characteristics of the study group in terms of vaginal (VD) and cesarean deliveries (CS) within 24 h, >24–48 h, and over 48 h.

	VD (*n* = 225)	CS (*n* = 66)	Total (*n* = 291)
**24 h**			
	145 (64.4%)	36 (54.5%)	181
**>24–48 h**			
	74 (32.9%)	26 (39.4%)	100
**>48 h**			
	6 (2.7%)	4 (6.1%)	10

**Table 7:** Characteristics of the study group in terms of operative deliveries within 24 h, >24–48 h, and over 48 h.

	Group (*n* = 12)
**24 h**	*n* = 11
Obstetrical forceps (F)	0 (0.0%)
Vacuum extractor (VE)	11 (100.0%)
**>24–48 h**	*n* = 1
Obstetrical forceps (F)	1 (100.0%)
Vacuum extractor (VE)	0 (0.0%)
**>48 h**	*n* = 0
Obstetrical forceps (F)	0 (0.0%)
Vacuum extractor (VE)	0 (0.0%)

**Table 8:** Characteristics of the group in terms of cesarean sections within 24 h, >24–48 h, and over 48 h.

	Group (*n* = 66)
**24 h**	*n* = 36
Fetal asphyxia	35 (97.2%)
Lack of labor progression	1 (2.8%)
**>24–48 h**	*n* = 26
Fetal asphyxia	22 (84.6%)
Lack of labor progression	4 (15.4%)
**>48 h**	*n* = 4
Fetal asphyxia	1 (25.0%)
Lack of labor progression	3 (75.0%)

**Table 9:** Comparative characteristics of the study group in terms of deliveries (vaginal (VD) and cesarean (CS)), oxytocin administration, intrapartum analgesia use, incidence of tachysystole, and duration of the first and second stages of labor within 24 h, >24–48 h, and over 48 h, stratified by BMI.

BMI	18.5–25 (*n* = 49)	25.5–30 (*n* = 125)	30–35 (*n* = 96)	35–40 (*n* = 16)	>40 (*n* = 5)	*p*-Value ^1^
**VD/CS 24 h**	35 (71.4%)	79 (63.2%)	58 (60.4%)	7 (43.8%)	2 (40.0%)	0.255
**VD/CS > 24–48 h**	13 (26.5%)	42 (33.6%)	34 (35.4%)	8 (50.0%)	3 (60.0%)	0.330
**VD/CS > 48 h**	1 (2.0%)	4 (3.2%)	4 (4.2%)	1 (6.3%)	0 (0.0%)	0.906
**VD 24 h**	30 (71.4%)	63 (63.6%)	48 (66.7%)	3 (37.5%)	1 (25.0%)	0.0325
**VD 24–48 h**	11 (26.2%)	33 (33.3%)	22 (30.6%)	5 (62.5%)	3 (75.0%)	0.3995
**VD > 48 h**	1 (2.4%)	3 (3.0%)	2 (2.8%)	0 (0.0%)	0 (0.0%)	0.9722
**CS 24 h**	5 (71.4%)	16 (61.5%)	10 (41.7%)	4 (50.0%)	1 (100.0%)	0.525
**CS 24–48 h**	2 (28.6%)	9 (34.6%)	12 (50.0%)	3 (37.5%)	0 (0.0%)	0.217
**CS > 48 h**	0 (0.0%)	1 (3.8%)	2 (8.3%)	1 (12.5%)	0 (0.0%)	0.377
**Oxytocin**	14 (28.6%)	46 (36.8%)	38 (39.6%)	9 (56.3%)	3 (60.0%)	0.255
**First stage (h)**						0.189
range	1.0–10.0	1.0–16.0	0.5–12.0	1.0–12.0	0.5–7.5	
median (IRQ)	3.0 (2.5)	4.0 (3.5)	3.5 (3.0)	4.0 (3.0)	6.5 (5.0)	
**Second stage (h)**						0.426
range	0.5–2.5	0.5–2.5	0.5–3.0	0.5–2.0	0.5–6.0	
median (IRQ)	0.8 (0.5)	1.0 (0.8)	0.5 (0.6)	1.0 (1.1)	1.0 (2.8)	
**Analgesia**						
Remifentanil	12 (24.5%)	38 (30.4%)	24 (25.0%)	7 (43.8%)	1 (20%)	0.5256
Epidural analgesia	18 (36.7%)	31 (24.8%)	28 (29.2%)	3 (18.8%)	3 (60.0%)	0.2252
**Tachysystole**	0 (0.0%)	2 (1.6%)	1 (1.0%)	2 (12.5%)	0 (0.0%)	0.0157

^1^ Chi- square; VD: vaginal delivery; CS: cesarean section.

**Table 10:** Comparative characteristics of the group in terms of time to regular contractions (TRC), time to pain onset (TPO), time to delivery (TD), blood loss (BL), perineal injuries (PI) and BMI.

BMI	18.5–25 (*n* = 49)	25.5–30 (*n* = 125)	30–35 (*n* = 96)	35–40 (*n* = 16)	>40 (*n* = 5)	*p*-Value ^1,2^
**TRC (h)**						0.087
mean (SD)	15.0 (12.3)	16.8 (11.1)	16.3 (11.2)	21.5 (13.5)	25.1 (10.1)	
range	1.0–61.5	0.5–52.0	0.5–57.5	1.0–55.0	15.5–39.0	
median (IRQ)	10.0 (20.5)	14.0 (16.0)	14.0 (16.0)	22.5 (18.3)	22.0 (15.0)	
95%CI	[11.4; 18.5]	[14.8; 18.8]	[14.1; 18.6]	[14.3; 28.7]	[12.6; 37.6]	
**TPO (h)**						0.077
mean (SD)	14.9 (12.4)	16.9 (11.1)	16.4 (11.2)	21.8 (13.7)	25.1 (10.1)	
range	1.0–61.5	0.5–52.0	0.5–58.0	1,0–55,0	15.5–39.0	
median (IRQ)	10.0 (20.0)	14.5 (16.0)	14.0 (16.0)	23.0 (18.3)	22.0 (15.0)	
95%CI	[11.4; 18.5]	[14.9; 18.8]	[14.1; 18.7]	[14.5; 29.1]	[12.6; 37.6]	
**TD (h)**						0.058
mean (SD)	19.3 (13.4)	21.9 (11.9)	21.5 (12.4)	26.4 (15.3)	31.5 (9.2)	
range	1.5–70.0	4.0–58.0	2.5–68.0	2.0–59.0	22.0–42.0	
median (IRQ)	14.5 (19.0)	19.0 (17.5)	19.0 (15.3)	28.0 (23.0)	30.0 (16.5)	
95%CI	[15.4; 23.2]	[19.8; 24.0]	[19.0; 24.0]	[18.3; 34.5]	[20.1; 42.9]	
**BL (mL)**						0.708
mean (SD)	289.8 (137.7)	314.0 (176.6)	308.3 (118.0)	340.6 (146.3)	320.0 (103.7)	
range	150.0–1 000.0	150.0–1 500.0	150.0–700.0	150.0–700.0	200.0–450.0	
median (IRQ)	250.0 (100.0)	250.0 (150.0)	250.0 (175.0)	300.0 (225.0)	300.0 (150.0)	
95%CI	[250.2; 329.3]	[282.6; 345.4]	[284.4; 332.2]	[262.7; 418.6]	[191.3; 448.7]	
**PI**						0.670
1	17 (51.5%)	33 (42.3%)	17 (30.9%)	4 (44.4%)	0 (0.0%)	
2	15 (45.5%)	43 (55.1%)	36 (65.5%)	5 (55.6%)	2 (100.0%)	
3	1 (3.0%)	2 (2.6%)	2 (3.6%)	0 (0.0%)	0 (0.0%)	

^1^ Kruskal–Wallis; ^2^ Ch-quadrat.

**Table 11:** Comparative characteristics of the group in terms of APGAR scores, blood gas analysis of umbilical artery (UA) and vein (UV) and BMI.

	18.5–25 (*n* = 49)	25.5–30 (*n* = 125)	30–35 (*n* = 96)	35–40 (*n* = 16)	>40 (*n* = 5)	*p*-Value ^1^
**1 min.**						0.088
range	6.0–10.0	6.0–10.0	6.0–10.0	8.0–10.0	9.0–10.0	
median (IRQ)	10.0 (0.0)	10.0 (0.0)	10.0 (0.0)	10.0 (0.5)	10.0 (0.0)	
**5 min.**						0.715
range	7.0–10.0	8.0–10.0	8.0–10.0	9.0–10.0	10.0–10.0	
median (IRQ)	10.0 (0.0)	10.0 (0.0)	10.0 (0.0)	10.0 (0.0)	10.0 (0.0)	
**10 min.**						0.623
range	7.0–10.0	10.0–10.0	8.0–10.0	10.0–10.0	10.0–10.0	
median (IRQ)	10.0 (0.0)	10.0 (0.0)	10.0 (0.0)	10.0 (0.0)	10.0 (0.0)	
**UA (pH)**						0.567
mean (SD)	7.2 (0.1)	7.3 (0.1)	7.3 (0.1)	7.3 (0.1)	7.2 (0.1)	
range	7.0–7.5	7.1–7.5	7.1–7.5	7.1–7.4	7.1–7.3	
median (IRQ)	7.2 (0.2)	7.2 (0.1)	7.3 (0.1)	7.3 (0.2)	7.3 (0.1)	
95%CI	[7.2; 7.3]	[7.2; 7.3]	[7.2; 7.3]	[7.2; 7.3]	[7.1; 7.4]	
**UV (pH)**						0.544
mean (SD)	7.3 (0.1)	7.3 (0.1)	7.3 (0.1)	7.3 (0.1)	7.3 (0.1)	
range	7.0–7.5	7.1–7.5	7.1–7.6	7.1–7.4	7.2–7.4	
median (IRQ)	7.3 (0.1)	7.3 (0.1)	7.3 (0.1)	7.3 (0.1)	7.3 (0.1)	
95%CI	[7.2; 7.3]	[7.3; 7.3]	[7.3; 7.3]	[7.3; 7.4]	[7.2; 7.4]	

^1^ Kruskal–Wallis.

**Table 12:** Correlations between selected parameters and BMI (R–correlation coefficient).

	R	*p*-Value ^1^
**First stage of labor**	0.08	0.189
**Second stage of labor**	−0.03	0.652
**Time to regular uterine contractions**	0.13	0.025
**Time to pain onset**	0.14	0.019
**Time to delivery**	0.15	0.013
**Blood loss**	0.07	0.203
**APGAR score at 1 min**	0.01	0.874
**APGAR score at 5 min**	0.03	0.596
**APGAR score at 10 min**	0.04	0.513
**pH UA**	0.10	0.095
**pH UV**	0.20	0.0005
**Initial Bishop score**	−0.07	0.241
**Number of Misoprostol doses**	0.15	0.013
**Bishop score after Misoprostol administration**	−0.17	0.005

^1^ Spearman.

## Data Availability

The data presented in this study are available on request from the corresponding author. The data are not publicly available due to privacy restrictions.
